# Identity disclosure and experience of discrimination of LGBT+ members of the veterinary professions in the UK: A mixed‐method approach using survey data

**DOI:** 10.1002/vro2.70015

**Published:** 2025-08-18

**Authors:** Mathew Hennessey, Bethany G. McAvery, Harry M. Warner, Megan Bird, Charlotte S. McCarroll

**Affiliations:** ^1^ Veterinary Epidemiology Economics and Public Health Group Department of Pathobiology and Population Sciences WOAH Collaborating Centre in Risk Analysis and Modelling, Royal Veterinary College London UK; ^2^ School of Veterinary Medicine University of Surrey Guildford UK

**Keywords:** discrimination, diversity, gender identity, identity disclosure, sexual orientation

## Abstract

**Background:**

Workplace and educational discrimination remain institutional issues, particularly for minoritised individuals, and can negatively affect performance and opportunity. This study sought to investigate the experience of discrimination and identity disclosure (being ‘out’) of lesbian, gay, bisexual, transgender and others (LGBT+) members of the UK veterinary professions and students.

**Methods:**

A mixed‐method approach was utilised to analyse 130 survey responses and examine associations between respondents’ identity disclosure (being ‘out’ or not) and other variables. Reflexive thematic analysis was conducted, based within minority stress theory, to generate themes to explain findings.

**Results:**

Over half the respondents reported having experienced or witnessed a range of discriminatory behaviours, and over half were not ‘out’ to all. When considering people's decisions around identity disclosure negative associations were detected for those who had witnessed discrimination and those who were unsure about antidiscrimination policy, and positive associations for those who felt supported at their place of work or study and by veterinary community in general. Three themes were generated that begin to explain respondents’ experiences.

**Limitations:**

Further research is required for institutions to know how our findings relate to their settings and to obtain additional in‐depth understanding of individuals exposure to discrimination.

**Conclusions:**

Our findings indicate that LGBT+ discrimination remains an issue for some veterinary professionals and students in the UK. For the profession to move towards a more equitable future, we suggest that a multifaceted approach is needed to bolster institutional support and the active promotion of equality, diversity and inclusion initiatives.

## INTRODUCTION

Discriminatory behaviour, harassment and victimisation of minoritised individuals based on sex, race, religion or belief, disability, age, marriage/civil partnership status, pregnancy, sexual orientation and gender reassignment status are prohibited in the UK by the Equality Act 2010.^1^ However, despite the presence of the Act, these behaviours are still recorded in workplaces.^2^ The British Veterinary Association (BVA) discrimination and voice surveys identified that the UK veterinary profession is not immune from workplace discrimination with 24%‒29% of respondents reporting experiencing or witnessing discriminatory behaviours.^3^ Despite responsibilities on places of work and study to adhere to the Equality Act, a survey of veterinary professionals and students by the Royal College of Veterinary Surgeons (RCVS) and British Veterinary Chronic Illness Support organisation found that most respondents had little or no knowledge of the legislation and little education or training was provided in workplaces.^4^

In recent years, research has evidenced discrimination among veterinary professionals, including students identifying as lesbian, gay, bisexual, transgender and others* (LGBT+). The British Veterinary LGBT+ (BVLGBT+) society found that 20% of students and 15% of professionals reported discrimination regarding their sexual orientation or gender identity.^5^ Similarly, experiences of discrimination were higher in non‐heterosexual individuals in the BVA surveys.^3^ These findings echo experiences of other students and professionals in the UK,^6^, ^7^, ^8^ including those in the UK medical profession,^9^ suggesting that while veterinary professions may be faring no worse, discrimination is still an area of concern.

Discrimination due to sexual orientation or gender identity has been shown to have a negative effect on mental health and wellbeing^10^ and a study of LGBT+ veterinary professionals and students in the United States and UK reported higher levels of suicidal ideation and attempted suicide compared with the general veterinary population.^11^ Most recently, a survey by Summers et al. examining perceptions of discriminatory behaviours experienced and witnessed by veterinary students undertaking clinical extra‐mural studies reported that over half of LGBTQ+ students had experienced or witnessed discrimination compared with a third of heterosexual students.^12^

Many LGBT+ individuals face a complex decision about whether to disclose their identity (being ‘out’ or not). Disclosure can foster authenticity and a sense of belonging,^13^, ^14^ reduce exposure to negative comments,^15^ and contribute to greater wellbeing.^16^ However, these studies also report negative consequences of disclosure including discrimination,^15^, ^16^ and lower career prospects and salary offers.^16^ The welfare survey by BVLGBT+ (2017) reported that almost a quarter of respondents were either only ‘out’ to close friends and colleagues or not ‘out’ at all. Again, this finding was similar to LGB employees across the UK,^6^ although that report does not include the experiences of transgender individuals. Fear of negative consequences can lead many to conceal their identity, which can cause stress, anxiety and a sense of disconnection.^17^ Consequently, these experiences make identity disclosure feel risky for many individuals.^17^, ^18^

Individual identity disclosure also has broader implications for organisations. A meta‐analysis of disclosure by Wax et al. found a positive correlation with employees being ‘out’ and job satisfaction and organisational commitment.^19^ Similarly, Tejeda found that the presence of non‐discrimination policies supported better relationships between management and teams.^15^ Conversely, workplace environments that are unsupportive of LGBT+ people have higher rates of staff turnover.^19^ Given the challenges currently facing recruitment in veterinary sectors,^20^, ^21^ it is prudent to consider how the profession can be as inclusive as possible, including understanding the experiences of LGBT+ individuals.

The aim of this study was to further investigate exposure to discrimination of LGBT+ members of the UK veterinary professions (including students), explore the consequence of such discrimination, and explain how this relates to identity disclosure.

## METHODS

### Survey design and implementation

The survey was modelled on two previous studies^3^, ^5^ to allow a degree of temporal comparison and contained questions regarding the participants demographics, ‘out’ status, exposure to discrimination (witnessing or experiencing), experience of support within the workplace and veterinary community and an opportunity to discuss anything else (Appendix 1). Participants were asked to report on their gender rather than their sex to more accurately capture their lived experiences and self‐identified identities, which are central to understanding the dynamics of LGBT+ discrimination and disclosure. There were 20 multiple‐choice questions and seven free‐text questions. Some questions were nested and only available to those that chose a particular answer. Eligible participants were those people working or studying in a related veterinary profession, based in the UK, over the age of 18 years and identified as LGBT+. Participants were recruited using convenience sampling. The survey, hosted on the online survey platform from the Joint Information Systems Committee (2025), was distributed through veterinary organisations, including BVA and BVLGBT+ via email communications to members and social media posts between 27 February 2023 and 31 March 2023.^22^ Participants were invited to enter a prize draw to win one of five £100 Amazon vouchers using a separate survey link at the end of the main survey where they could leave an email while maintaining anonymity of their answers.

### Data management

Survey responses were extracted into MS Excel, after which text from the open‐ended questions (Q9, 10, 11, 16, 17 and 18) was copied into MS Word for data familiarisation and further analysis. The 172 survey responses were assessed using our eligibility criteria and to examine data for fraudulent responses at the level of the questionnaire using criteria described by Teitcher et al. for inconsistent and improper answers.^23^ Consequently, 130 responses were used in the final analysis (Table 1).

**TABLE 1:** Exclusion criteria for responses not used in analysis.

Exclusion criteria	Number of responses excluded	Number of responses included
Initial responses (*n* = 172)		
Main place of work is not in the UK	4	168
Does not identify as LGBT+	30	138
Irregular computer information (repeated sequential entries during the night)	8	130

Abbreviation: LGBT+, lesbian, gay, bisexual, transgender and others.

### Data analysis

#### Quantitative analysis

Fisher's exact test^24^ was used to assess the difference in ‘out’ status between groups within the sexual orientation and gender identity variables reported in the survey. A Kruskal‒Wallis test^25^ was used to examine associations between participant's reported identity disclosure status and their responses to several variables reported in the survey. These variables were (1) respondent's exposure to discrimination (witnessing or experiencing), (2) their sense of support in the workplace or place of study, (3) their awareness of anti‐discrimination policies, and (4) their sense of feeling supported by the veterinary community. The Kruskal‒Wallis test was selected because the data were non‐parametric and the categorical response variables could classified as ordinal for the analysis. A significance level of 0.05 was used to determine statistical significance. All statistical analyses were conducted using SPSS (v.29).

#### Qualitative analysis

Social theory can help explain the relationship between stress and the experiences of LGBT+ individuals and include (but is not limited to) stress theory, intersectionality theory, social constructionism, queer and feminist theory, and social identity theory. We based our analysis within Meyer minority stress theory framework which they suggest can be used to explain how ‘stigma, prejudice and discrimination create hostile and stressful environment[s]’ (p. 1) and has been used to explain the experiences of LGBT+ people.^26^

**FIGURE 1:**
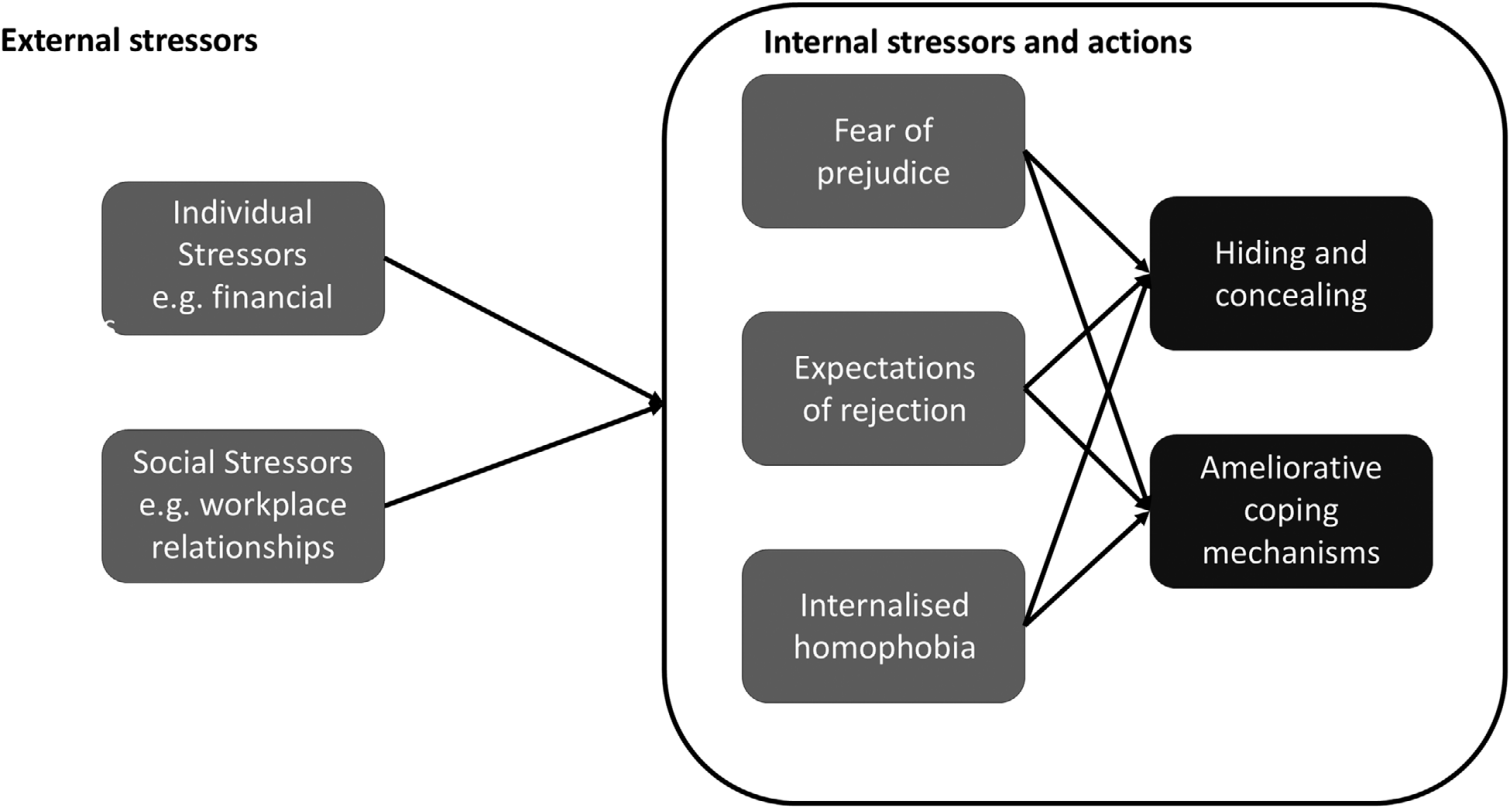
Conceptual framework for analysing lesbian, gay, bisexual, transgender and others (LGBT+) related stress (adapted from Meyer, 2003).

Here, Meyer describes stressors as distal (or external) and proximal (or internal) (Figure 1). External stressors due to marginalisation are those that are observable to a person, objective, and may include physical or verbal abuse or employment discrimination. Internal stressors include fears about discrimination and rejection and extend the internalisation of societal phobias.

To explain our findings, we conducted reflexive thematic analysis, as described by Braun and Clark^27^ and Hennessey and Barnett^28^ on free‐text responses. Here, data were coded (by M.H.) in NVivo (v.11) using a deductive approach based on a relativist ontological and constructivist epistemological position†. Themes generated were refined during discussion between the first (M.H.) and last authors (C.M.).

## RESULTS

### Quantitative results

#### Descriptive summary

**FIGURE 2:**
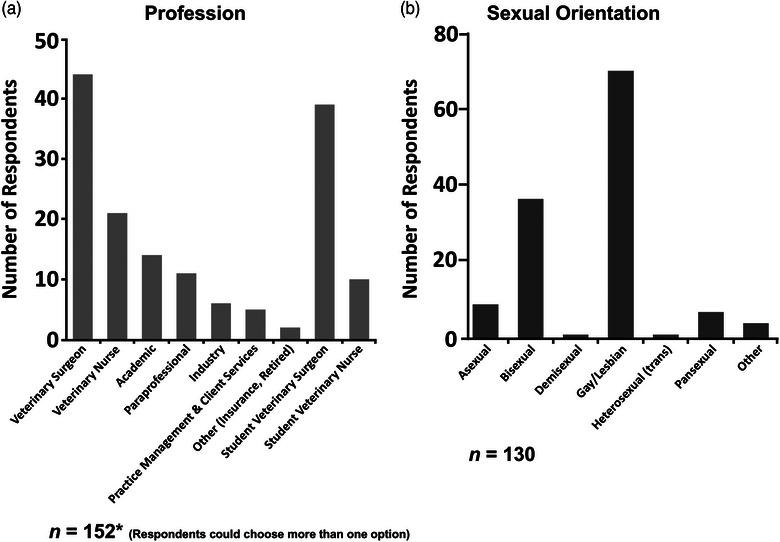
Survey respondents professions (a) and sexual orientation (b).

A total of 130 responses met the inclusion criteria. Respondents’ roles were veterinary surgeons (33.8%), veterinary medical students (30.0%), veterinary nurses (16.2%) and nursing students (7.7%). Other categories included academics, veterinary paraprofessionals, those working in industry (such as for pharmaceutical companies), practice management, client services and insurance (Figure 2A). Respondent genders were female (48.5%), male (43.8%), non‐binary (5.4%) and gender fluid (2.3%). Almost one in eight of respondents reported that their gender did not match that assigned at birth either all or some of the time.

Reported sexual orientations were gay/lesbian (54.6%) and bisexual (28.5%), with others identifying as asexual, pansexual, queer, demisexual and heterosexual (trans) (Figure 2B and Table 2).

**TABLE 2:** Respondents ‘out’ status differentiated by sexual orientation and gender identity for students and non‐students.

	‘Out’ to all	‘Out’ to majority	‘Out’ to close friends/colleagues	‘Out’ to none	Total (%)
Sexual orientation					
Asexual	2		4	3	9 (6.9)
Bisexual	9	12	12	4	37 (28.5)
Demisexual				1	1 (0.8)
Gay/lesbian	49	10	10	2	71 (54.6)
Heterosexual (trans)		1			1 (0.8)
Pansexual	3	2	2		7 (5.4)
Queer		1	3		4 (3.1)
Total (%)	63 (48.5)	26 (20)	31 (23.8)	10 (7.7)	
Gender identity					
Matches that at birth	61	21	23	9	114 (87.7)
Does not always match that at birth	2	5	8	1	16 (12.3)

Almost half of respondents (48.5%) said they were ‘out’ to all people, 20% said they were ‘out’ to most people, 23.8% were only ‘out’ to close friends and colleagues, while 7.7% said they were ‘out’ to no‐one. Table 2 provides a summary of people's ‘out’ status differentiated by sexual orientation and whether their gender matches that assigned at birth. The survey also included an open‐ended question to provide an opportunity for respondents to explain decisions around their identity disclosure, which we explore further in the second part of our analysis.

The characteristics described across Table 2 were reduced to binary variables for sexual identity (gay/lesbian or other) and ‘out’ status (‘out’ to most/all or not) to allow a Fisher's exact test to be performed. These results indicate that there was a significant difference between identifying as gay/lesbian or another sexual orientation and being ‘out’ to most or all people (*p* = 0.0001). Here, there were fewer respondents with a sexual orientation different from gay/lesbian being ‘out’ to all or most than those identifying as gay/lesbian. Similarly, for gender identity, there was a significant difference between people who reported having a gender that matches that assigned at birth (cis‐gendered) and those who did not (trans‐gendered) and being ‘out’ to all or most people (*p* = 0.0408). Here, there were fewer respondents who said that their gender did not always match that assigned at birth being ‘out’ to all or most people rather than cis‐gendered people.

Overall, 55.4% of respondents reported having experienced or witnessed behaviour, which could be considered discriminatory related to sexual orientation and/or gender identity. Incidents reported by students occurred at similar frequencies across places of study, work or placement, while incidents reported by non‐students primarily occurred at their place of work. Of those people who reported exposure to discriminatory behaviour, most (72.2%) provided a description of the event in the survey, the analysis of which we provide in the subsequent part of our analysis.

#### Association of ‘out’ status with other variables

When respondents’ ‘out’ status was examined against reports of discrimination (Table 3), a significant difference was detected for those people who reported witnessing discrimination (*p* = 0.014). There was a greater proportion of people who reported only being ‘out’ to close friends or colleagues or not ‘out’ at all selecting ‘witnessed’ to the question ‘have you experienced or witnessed any discriminatory behaviour?’.

**TABLE 3:** Association of sexual orientation and/or gender identity‐based discrimination with ‘out’ status.

	Reported discrimination			
	No	Experienced	Witnessed	Both
Kruskal‒Wallis H	3.090	3.144	10.651	2.750
Degrees of freedom	3	3	3	3
Significance	0.378	0.370	0.014^*^	0.432

* *p* < 0.05.

Examining respondents ‘out’ status against their sense of being supported in their place or work or study (Table 4), a significant difference was detected for those people who reported that they always felt supported (*p* = 0.02). Here, there was a greater proportion of people who reported being ‘out’ to all selecting ‘yes always’ to the question ‘Do you feel you get informal support from your place of work or study in terms of your sexual orientation and/or gender identity?’.

**TABLE 4:** Association of feeling informally supported by workplace or place of study with ‘out’ status.

	Sense of being supported			
	No	Not sure	Yes sometimes	Yes always
Kruskal‒Wallis H	3.185	5.117	3.949	9.817
Degrees of freedom	3	3	3	3
Significance	0.364	0.163	0.267	0.020^*^

* *p* < 0.05.

When examining respondents’ awareness of formal discrimination policies (Table 5), a significant difference was detected for those people who were unsure. There was a greater proportion of people who reported not being ‘out’ at all or only ‘out’ to close friends and colleagues selecting ‘not sure’ when asked if they were aware of a workplace discrimination policy.

**TABLE 5:** Association of awareness of formal workplace support policies with ‘out’ status.

	Awareness of workplace discrimination policy		
	No	Not sure	Yes
Kruskal‒Wallis H	2.309	9.207	1.606
Degrees of freedom	3	3	3
Significance	0.511	0.027^*^	0.658

* *p* < 0.05.

Examining respondents ‘out’ status against their sense of being supported by the veterinary community (Table 6), a statistical difference was detected for those who reported feeling always supported. Here, there was a greater proportion of people who were ‘out’ to all selecting ‘yes always’ when asked if they felt supported by the veterinary community.

**TABLE 6:** Association of feeling supported by the veterinary community with ‘out’ status.

	Sense of being supported by the veterinary community			
	No	Not sure	Yes sometimes	Yes always
Kruskal‒Wallis H	7.000	6.005	5.498	12.343
Degrees of freedom	3	3	3	3
Significance	0.072	0.111	0.139	0.006^*^

* *p* < 0.05.

### Qualitative analysis

In this section, we explore what exposure to discrimination means for LGBT+ professionals and students and how people navigate decisions around identity disclosure.

#### External stressors: summary of types of discrimination

As reported in the previous section, of the 55.4% of respondents (*n* = 72) who reported exposure to discriminatory behaviour (witnessing or experiencing), most (72.2%) provided additional detail describing the nature of this exposure, which we describe here. These ranged from, at the most extreme and concerning, incidents of physical violence, reports of LGBT+ phobias, inappropriate questioning regarding sexual preferences and acts, ‘outing’ people without their permission, to various types of microaggressions.

The topic of pronoun use was raised by several people who reported incidences of colleagues making fun of alternative pronouns (e.g., they/them) or saying these were unnecessary. Several people mentioned times when people had been misgendered, with some, such as the following respondent, questioning whether this was an intentional act or an accident:

‘Someone I knew was misgendered a few times, after being informed what their pronouns were. It was somewhere between on purpose and an accident. I don't think the person was being malicious, but I don't think they were trying to use the correct pronouns’. (ref. 107095807)

In addition to concerns around pronoun use, several people reported exposure to anti‐trans comments. Two respondents mentioned how colleagues made comments on the appearance of trans clients, while two others described how colleagues talked negatively about trans people in the context of inclusion within sport.

Colleagues or clients using the word ‘gay’ in a negative manner were also reported by several people. Exposure (witnessing or experiencing) to this ranged from colleagues using phrases such as ‘that's so gay’ to describe a negative event (ref. 107997232) to clients describing their dog as gay during a clinical consultation because the animal showed fearful behaviour.

#### Internal stressors: summary of fears of discrimination

Our study findings indicate that just over half of respondents (51.5%) were not ‘out’ to all people, some choosing to hide their LGBT+ identity from specific people, some only telling close friends and colleagues, and some not being ‘out’ to anyone. The majority of respondents (69.2%) provided additional information explaining their decision around identity disclosure. Many of these explanations for not being ‘out’ to all people described a fear of an LGBT+ identity negatively affecting their education or career. Some people described this as a fear of judgement by others, often those in positions of seniority, resulting in perceptions of reduced employability and career options, or like this student, being left out of educational opportunities:

‘If I expect [clinicians] to not be very open, I will not come out either due to fear of not being given as much responsibility and not being able to learn as much on the rotation’. (ref. 1067002972)

Many people held a perception that large animal (or ‘farm’) veterinary work could expose them to more discrimination than other areas. Here, a concern expressed by several people, including the following person, was that older farm clients would hold more conservative and traditional views on identity and so there existed a greater fear of a negative experience:

‘I work in large animal practice, and I wouldn't be open to all of our clients. Some are elderly farmers who do not have the same concept of inclusion’. (ref. 106848409)

However, as one respondent acknowledged, this perceived fear of a negative identity reaction could be due to stereotypical views of older farmers rather than an actual reality.

#### Responses to minority stress: thematic analysis

We generated three key themes which begin to explore how LGBT+ individuals respond to minority stress in the veterinary profession.

##### Theme 1: Searching for a haven to ameliorate identity risk

When commenting on their decision whether to be ‘out’ in their place or work or study, most people mentioned the need to feel safe, comfortable and to be in a supportive environment. However, two people did comment that their reason for not being ‘out’ was that it ‘had never come up in conversation’ (ref. 108085029) or that their ‘sexual orientation is not relevant’ to their work (ref. 106847517).

The desire for a safe and supportive environment suggests that, should these criteria not be met then conflict about identity disclosure will arise. Various factors contribute to this idea of safety, including being around other LGBT+ colleagues, having good relationships with peers and leaders, making friends with peers, and trust in colleagues. All these factors contribute to an ability of an individual to assess the possible reactions to identity disclosure. Given this, it is unsurprising that some people indicated they felt more comfortable in a small team of people, while some found large groups, or like this student, uncertain situations, more challenging to navigate:

‘I feel safe in my school environment to share that I am part of the LGBT+ community without much fear of retribution. There is still some fear when entering a new workplace and I chose not to share until I feel comfortable enough’. (ref. 106779960)

Many people who reported being ‘out’ with all people described their places of work or study in positive terms including ‘inclusive’, ‘diverse’, or like the following person ‘supportive’ and ‘open’:

‘It is a supportive and open workplace, and I haven't felt the need to be closeted. I am out to everyone at work as I felt comfortable enough to speak openly about my partner’. (ref. 107564992)

Several people, who reported only being ‘out’ with close friends or to no‐one described either not being close enough to people to share identity information with them, or like this professional, explained how past negative experiences affected their decision to disclose:

‘[I] would not be comfortable sharing [my identity] with people I am not close to. [I] have known some to make discriminatory comments or observations, so do not want that attention’. (ref. 106846923)

This theme indicates that some veterinary professionals and students have to collect data from their surroundings on an ongoing basis before making a decision about identity disclosure.

##### Theme 2: Hiding away and not always being ‘out of the closet’

As introduced in the previous theme, many survey respondents reported a hesitancy to disclose their identity in uncertain situations, or in settings of previous negative experience. Consequently, this hesitancy can lead to tempering behaviour ranging from the removal of LGBT+ symbols (e.g., badges and lanyards) from their person, acting differently (e.g., being ‘less outwardly effeminate and playful’; ref. 106804851), or, like the following person, not challenging assumptions of heteronormativity:

‘I never correct people when they assume that my partner is of the opposite gender because I am single. I feel like they may dislike me for bringing up my homosexuality when there is no explicit need’. (ref. 106759606)

Several people mentioned how they avoided conversations with peers that engaged with topics relating to relationships or family, or, like the following person, were hesitant of talking about engagement with LGBT+ events:

‘[I avoid] conversations about relationships/dating (unless it's just others talking about their experiences) where I could be asked stuff. Also, although I'll happily talk supportively about the LGBTQ+ community as a whole, I may not engage in a discussion where I mention personally going to a pride festival in case anyone in the environment isn't supportive of that’. (ref. 106698211)

For some people, conflict exists between the stress of hiding away and the uncertainty associated with identity disclosure:

‘I got tired of hiding. It wasn't doing me any good. I decided I would rather be myself and face the challenges that came with that (explaining pronouns, not having a correct bathroom etc.) than the dysphoria’. (ref. 106985304)

This theme highlights the challenge faced by individuals experiencing degrees of perceived environment safety and are thus required to temper behaviour and hide their identity away.

##### Theme 3: A duty to be visible to represent the community for others

Several people mentioned that part of their decision to be ‘out’ in their place of work of study was related to a duty they felt to be a representative of the LGBT+ community to others. For example, the following professional talked about the need to be visible to students:

‘Working at a university and being involved with some teaching I feel I have a duty to be a visible role model to students’. (ref. 10611220)

Visibility in places or work or study took various forms, from engaging in relevant conversations, wearing LGBT+ symbols, such as pride pins and lanyards, to adding pronoun signatures onto email signoffs. However, even those in extremely visible roles, such as the following person in an equality, diversity and inclusion (EDI) position, reported times when such visibility was removed:

‘I hold an EDI position related to LGBTQI+ matters, and I choose to use an abbreviated version of my email signature when contacting unknowns outside of the organisation’. (ref. 107246681)

As mentioned in the first theme, one of the factors used by survey respondents to judge whether an environment was safe of not was the presence of other LGBT+ people. Thus, having individuals fulfilling a duty to be visible appears an important aspect of creating a supportive environment for those who may be less confident about identity disclosure.

## DISCUSSION

The findings of this mixed‐methods study highlight the experience of discrimination faced by LGBT+ veterinary professionals and students in the UK and how this discrimination can influence identity disclosure.

The survey shows a snapshot of perspectives, demonstrating how a proportion of LGBT+ individuals within the veterinary professions have experienced or witnessed discrimination. The types of LGBT+ discrimination were similar to experiences of veterinary professionals and students in the United States and the UK,^29^ which included various types of microaggressions, people being outed, and in the extreme, threats of violence. Our finding that 55.4% of respondents reported exposure to discrimination is much higher than previously reported in the UK veterinary professions and students by BVLGBT+ in 2017^5^ and in a study by Witte et al. who describe how one‐third of veterinarians and veterinary students, from the United States and UK, reported exposure to discrimination related to their LGBT+ identity.^11^ This difference could reflect a concerning increase in exposure to discrimination. Indeed, in England and Wales, records of hate crimes related to sexual orientation or gender identity increased by 107% between 2017 and 2023.^30^ However, the increased exposure to discrimination in our survey may be partially explained by the previous BVLGBT+ survey only asking people if they had experienced discrimination and not witnessed it.^5^ Either way, our findings underscore an ongoing presence of discriminatory behaviour in professional and educational settings. The qualitative data further enrich this understanding by illustrating the range and nature of discriminatory experiences. The diversity of these experiences suggests that discrimination is not confined to overt hostility but also encompasses subtler forms of exclusion and marginalisation.

The impact of such discrimination is multifaceted. The fear of negative repercussions, particularly regarding career progression and educational attainment, was a key concern among participants. These findings align with broader literature on workplace discrimination, which highlights the detrimental effects of fear of disclosure on professional development and mental wellbeing.^16^, ^19^, ^29^, ^31^ In a similar study of discrimination of LGBT+ veterinary professionals and students in the United States and the UK, Witte et al.^11^ and Kramper et al.^29^ describe the range of negative emotional outcomes of such experiences, including isolation, loneliness, anxiety, depression and on occasion suicide ideation. Kramper et al. reported that one coping tactic for professionals is to change jobs and noted how this avenue is not often possible for students who face barriers in moving institutions.^29^

Our survey found around one‐third of participants were either not ‘out’ or only ‘out’ to close colleagues or friends. This finding is similar to that previously reported for members of the veterinary professions in the UK in 2017 (23% of people not ‘out’ or only ‘out’ to close colleagues or friends^5^), veterinarians and veterinary students in the United States and the UK,^29^ and consistent with reports in wider UK society.^6^ These findings suggest that there remains significant hesitancy to be fully open in professional veterinary settings which our quantitative and qualitative analyses provide further insight into.

Considering sexual orientation and gender identity within the context of identity disclosure, our finding that people were more likely to be ‘out’ to most or all people if they were either cis‐gendered or identified as gay or lesbian reflects wider trends. In the UK, the LGBT+ organisation, Stonewall reports that 38% of bisexual people are not ‘out’ to anyone at work, compared to 7% of gay men and 4% of lesbians.^32^ Similarly, a report of trans people's experiences at work in the UK found that only 41% of respondents felt that their workplace was trans inclusive.^33^ This indicates that additional resources may need to be directed towards specific marginalised groups within the broader LGBT+ community.

Results from our analyses provide further context for patterns of identify disclosure. The themes we generated illustrate the tension between the need for safety and the desire for representation. Many LGBT+ individuals actively seek out safe spaces where they can be authentic without fear of discrimination. Mink et al. describe the potential negative health outcome of such ‘hypervigilance’ with LGBT+ individuals in a predominately heteronormative settings experiencing elevated and chronic stress.^34^ Conversely, others choose to conceal their identity. Indeed, one of the results from our statistical analysis indicates that a greater proportion of people who are ‘out’ to only close colleagues or to no one reported witnessing more discrimination than those who are ‘out’ to most or all. This may be due to those individuals seeking to hide their identity being hypervigilant to the experiences of others as they monitor the perceived safety of their environment. While it is possible that some people may not disclose their identity, despite a perception of a safe environment, due to being more private or not seeing this as relevant, this was generally not reflected across the responses from our study. Our findings indicate that people want to be ‘out’ and those who do not disclose their identity make this choice because they perceive their professional environment as unwelcoming and want to avoid potential negative repercussions. This aligns with existing models of identity management, such as the concept of ‘covering’ described by Yoshino, where individuals downplay aspects of their identity to fit in.^35^ However, despite this being a strategy to fit in, our findings indicate one consequence of not being ‘out’ can be the avoidance of personal conversations with colleagues. Consequently, this avoidance may act as a barrier for colleagues to get to know each other and form the type of peer relationships deemed necessary for an environment to be considered safe, as we describe in theme one ‘searching for a haven’. Conversely, some individuals feel a responsibility to represent the community and advocate for change, despite the potential risks associated with doing so. In their study on the effects of discrimination, Kramper et al. noted the need and desire of those LGBT+ veterinarians and veterinary students who strive to build a community and work with those like‐minded individuals to create an inclusive environment.^29^ On a positive note, our findings that a greater proportion of those people who are ‘out’ to all or most felt a sense of being supported by their place or work or study, or by the veterinary community than those who are not out, indicates that the decision to disclose an LGBT+ identity can be worthwhile for many people. Thus, our study highlights the complex and ongoing negotiations people may experience that engage with issues such as personal safety and collective progress, and where LGBT+ visibility can contribute to increased acceptance but also expose individuals to uncertainty.

The reluctance of many LGBT+ individuals to be ‘out’ at work or in education has broader implications for the veterinary profession. A lack of visibility can contribute to a cycle in which LGBT+ identities remain marginalised, reinforcing the perception that the profession is not fully inclusive. As highlighted in the second theme, ‘hiding away and not always being out’, it is concerning that there may be times when some individuals decide to hide away their LGBT+ identity, thus potentially weakening the overall cohesiveness of an inclusive environment. Additionally, the stress and emotional labour associated with managing identity disclosure may negatively impact job satisfaction, retention and overall wellbeing.^13^, ^16^, ^26^

Creating a more supportive and inclusive environment requires structural and cultural changes. While EDI policies and initiatives are important, their effectiveness is contingent on effective communication, as well as implementation and enforcement. Recent research suggests that additional work is needed to improve education and training regarding anti‐discrimination legislation in the veterinary community.^4^ Our finding that a greater proportion of respondents who were only ‘out’ to close colleagues or not ‘out’ at all being unsure of discrimination policies than those who are ‘out’ to most or all, indicates a need for institutions to always be open and transparent about such policies, not just when someone of a minoritised identity is present. In the UK, the RCVS provides guidance on veterinary employers’ obligations under the Equality Act (2013), setting expectations on how veterinary professionals and students should be treated. We suggest that resources and training on LGBT+ inclusivity, such as the BVA's ‘good workplace resources’ and microaggression posters,^36^ should be integrated into both veterinary education and workplace policies. Furthermore, mentorship programmes could be developed and LGBT+ networks within the profession, such as BVLGBT+,^37^ signposted to provide support for individuals navigating identity disclosure and discrimination. Furthermore, we argue there is a need to move beyond equality and engage with the concept of equity to recognise variation in individual needs and circumstances to allocate resources accordingly and ensure fair outcomes and equal access to opportunity. These debates are now particularly salient given that institutional efforts to undermine EDI work taking place in certain parts of the world, such as the current anti‐EDI agenda in the United States,^38^ and bans on gender studies and Pride marches by the Hungarian government, for example.^39^, ^40^ We also note that additional action to support trans members of veterinary professions in the UK is now needed given the 2025 Supreme Court ruling that ‘sex’ means biological sex^41^ to ensure that the Equality Act prevents unlawful discrimination based on gender reassignment.

### Limitations

By generating data through a survey, the lack of interaction between researchers and respondents removes the opportunity for deeper probing of lines of enquiry. However, survey data have the advantage that people can respond anonymously and in their own time, and potentially a greater number of people can participate. Given this, we suggest that, first, institutions should seek to know whether their people have similar experiences to what we describe. Second, further qualitative studies, such as in‐depth interviews or life history accounts, are needed to examine more complex areas of enquiry, such as the effect of internalisation of phobias^26^ and self‐stigma on mental health,^42^ how LGBT+ lived experiences intersect with other marginalised identities (such as race, ethnicity, neurodiversity), and to probe into the institutional and contextual factors that impact people's decisions regarding identity disclosure.

## CONCLUSION

The findings from this study indicate that discrimination remains an issue within the UK veterinary profession, affecting some LGBT+ individuals’ willingness to disclose a marginalised identity and their overall sense of support. The themes we have generated illustrate ways in which many individuals manage their identity when faced with an uncertain environment and their responses to potential risk of disclosure. We suggest that addressing such issues requires a multifaceted approach that includes institutional support, cultural change and the active promotion of inclusivity. By fostering an environment where LGBT+ veterinary professionals and students feel safe, supported and valued, the profession can move towards a more equitable and diverse future.

## AUTHOR CONTRIBUTIONS

Mathew Hennessey contributed to conceptualisation, data curation, formal analysis, methodology, project administration, validation, visualisation, writing—original draft, and review and editing. Bethany G. McAvery contributed to data curation, formal analysis, investigation, and review and editing. Harry M. Warner contributed to data curation, formal analysis, investigation, and review and editing. Megan Bird contributed to data curation, formal analysis, investigation, and review and editing. Charlotte S. McCarroll contributed to conceptualisation, data curation, formal analysis, funding acquisition, investigation, methodology, project administration, supervision, validation, visualisation, writing the original draft, and review and editing. All authors have approved the final version of the manuscript for publication.

## CONFLICTS OF INTEREST

The authors declare they have no conflicts of interest.

## ETHICS STATEMENT

Ethical approval was obtained from University of Surrey's ethics committee (ref. FEO FHMS 22‐23 033 EGA). All people were asked to provide their consent to participate by ticking a check box on the survey after reading an ethics statement. A statement signposting participants to relevant support services (Vet Life, LGBT Foundation and The Samaritans) was also provided on the information sheet.

## Supporting information



Supporting Information

## Data Availability

Data are available upon request due to privacy/ethical restrictions. The data that support the findings of this study are available upon request from the corresponding author. The data are not publicly available due to privacy or ethical restrictions.

## Footnotes

* Here, ‘others’ or ‘+’ refers to identities not covered by LGBT, including (but not limited to) asexual, demisexual, intersex, pansexual, queer.

† This positioning was adopted to recognise that social reality is not fixed but is instead shaped by individual experience and perspectives, and so the reality we present here is constructed from interactions between the research team, survey data and theoretical framing
