# Supplementary material for: Identity disclosure and experience of discrimination of LGBT+ members of the veterinary professions in the UK: A mixed‐method approach using survey data
Source: Vet Rec Open. 2025 Aug 18;12(2):e70015. doi: 10.1002/vro2.70015 (PMC12358733; doi:10.1002/vro2.70015)
Supplement: Supplementary file 1 — Supporting Information [file VRO2-12-e70015-s001.docx]

## Appendix 1. Survey questions

Survey title: ‘British Veterinary LGBT+ Welfare and Discrimination Survey’.

1. I have read and understood the information provided by the ‘Participant Information’ and ‘Informed Consent’ pages and consent to taking part in this study
   1. Yes
   2. No
2. What is your role within the veterinary profession? (Tick all that apply)
   1. Veterinary Surgeon - Clinical
   2. Veterinary Surgeon - Student
   3. Veterinary Nurse - Clinical
   4. Veterinary Nurse – Student
   5. Academia
   6. Government
   7. Industry
   8. Veterinary paraprofessional (Veterinary Care Assistant, Pharmacist, Technician etc)
   9. Veterinary practice management
   10. Retired
   11. Prefer not to say
   12. Other
       1. If you selected Other, please specify:
3. Where is/was your main place of work?
   1. UK
   2. Overseas RCVS recognised
   3. Overseas non-RCVS recognised
4. What is your age (to nearest whole year)?
   1. 18-24
   2. 25-34
   3. 35-44
   4. 45-54
   5. 55-64
   6. 65-74
   7. 75 or more
   8. Prefer not to say
5. What term best describes your gender?
   1. Male
   2. Female
   3. Non-binary
   4. Genderfluid
   5. Prefer not to say
   6. Other
      1. If you selected Other, please specify:
6. Does your gender match that assigned at birth?
   1. Yes
   2. No
   3. Sometimes
   4. Prefer not to say
7. What term best describes your sexual orientation?
   1. Heterosexual
   2. Homosexual
   3. Bisexual
   4. Pansexual
   5. Asexual
   6. Prefer not to say
   7. Other
      1. If you selected Other, please specify:
8. Are you out at work? Which statement best describe your situation?
   1. I am out to all people
   2. I am out to the majority of my colleagues but there are some people I chose not to tell
   3. I am out only with close friends/colleagues
   4. No, I am not out at work
   5. Prefer not to say
9. Why are you out to all/some/none? (Optional)
10. Are there any scenarios you avoid due to fear of discrimination? (Optional)
11. Have you personally experienced or witnessed any behaviour that you think was or could be discriminatory related to sexual orientation and/or gender identity?
    1. No
    2. Yes/not sure
       1. How have you experienced or witnesses discriminatory behaviour?
          1. Personally experienced
          2. Witnessed occur to someone else
          3. Both experienced and witnessed
       2. How many incidents of discriminatory behaviour?
          1. 1
          2. 2-4
          3. 5+
       3. When did the most recent discriminatory behaviour(s) occur?
          1. Within last 12 months
          2. 1-2 years ago
          3. 3-5 years ago
          4. 5+ years ago
       4. What was the subject of the discriminatory behaviour(s)? (Tick all that apply)
          1. Sexual orientation
          2. Gender Identity
          3. Prefer not to say
          4. Other
             1. If you selected Other, please specify:
       5. Where did the discriminatory behaviour(s) take place? (Tick all that apply)
          1. At place of work
          2. Out on a call
          3. At place of study
          4. Out on placement
          5. Prefer not to say
          6. Other
             1. If you selected Other, please specify:
       6. What was the role of the perpetrator of the discriminatory behaviour(s)? (Tick all that apply)
          1. Veterinary Surgeon - supervisor
          2. Veterinary Surgeon – colleague
          3. Veterinary Surgeon - subordinate
          4. Veterinary Nurse – supervisor
          5. Veterinary Nurse – colleague
          6. Veterinary Nurse - subordinate
          7. Practice Manager
          8. Student – colleague
          9. Student - seeing practice
          10. Member of the public
          11. Veterinary paraprofessional
          12. Reception/Administrative staff
          13. Prefer not to say
          14. Other
              1. If you selected Other, please specify:
       7. What form did you perceive the discriminatory behaviour take? (Tick all that apply)
          1. Discriminatory comment(s) from ignorance
          2. Discriminatory comment(s) from malice
          3. Inappropriate questioning from ignorance
          4. Inappropriate questioning from malice
          5. Microaggression
          6. Physical assault
          7. Hostile atmosphere
          8. Other
             1. If you selected Other, please specify:
       8. Please give a brief description of the discriminatory behaviour(s) (Optional)
12. Do you feel you get informal support from your place of work/study in terms of your sexual orientation and/or gender identity?
    1. Yes – always
    2. Yes – sometimes
    3. No
    4. Not sure
    5. Prefer not to say
13. Are you aware of a formal policy supporting you at your workplace?
    1. Yes
       1. Do you believe that the formal policy at your workplace is followed?
          1. Yes
          2. No
          3. Not sure
          4. Prefer not to say
    2. No
    3. Not sure
    4. Prefer not to say
14. Please feel free to expand on any support you feel you are or are not getting from your place of work/study (Optional)
15. Do you feel supported within the veterinary community at large in terms of your sexual orientation and/or gender identity?
    1. Yes – always
    2. Yes – sometimes
    3. No
    4. Not sure
    5. Prefer not to say
16. Please feel free to expand on any support you feel you are or are not getting from within the veterinary community at large (Optional)
17. As a LGBTQ+ community what support, resources or guidance would you like to see from BVLGBT+? (Optional)
18. Do you have positive or negative experiences you would like to share? (Optional)
